# Mucin Glycans: A Target for Cancer Therapy

**DOI:** 10.3390/molecules28207033

**Published:** 2023-10-11

**Authors:** Lingbo Sun, Yuhan Zhang, Wenyan Li, Jing Zhang, Yuecheng Zhang

**Affiliations:** 1 Medical College of Yan’an University, Yan’an University, Yan’an 716000, China; lingbosun@yau.edu.cn (L.S.); z13201471702@outlook.com (Y.Z.); lwy2261344932@outlook.com (W.L.); 2 Key Laboratory of Analytical Technology and Detection of Yan’an, College of Chemistry and Chemical Engineering, Yan’an University, Yan’an 716000, China

**Keywords:** mucin, glycosylation, cancer, MUC1, targeted therapy

## Abstract

Mucin glycans are an important component of the mucus barrier and a vital defence against physical and chemical damage as well as pathogens. There are 20 mucins in the human body, which can be classified into secreted mucins and transmembrane mucins according to their distributions. The major difference between them is that secreted mucins do not have transmembrane structural domains, and the expression of each mucin is organ and cell-specific. Under physiological conditions, mucin glycans are involved in the composition of the mucus barrier and thus protect the body from infection and injury. However, abnormal expression of mucin glycans can lead to the occurrence of diseases, especially cancer, through various mechanisms. Therefore, targeting mucin glycans for the diagnosis and treatment of cancer has always been a promising research direction. Here, we first summarize the main types of glycosylation (O-GalNAc glycosylation and N-glycosylation) on mucins and the mechanisms by which abnormal mucin glycans occur. Next, how abnormal mucin glycans contribute to cancer development is described. Finally, we summarize MUC1-based antibodies, vaccines, radio-pharmaceuticals, and CAR-T therapies using the best characterized MUC1 as an example. In this section, we specifically elaborate on the recent new cancer therapy CAR-M, which may bring new hope to cancer patients.

## 1. Introduction

**Figure 1:**
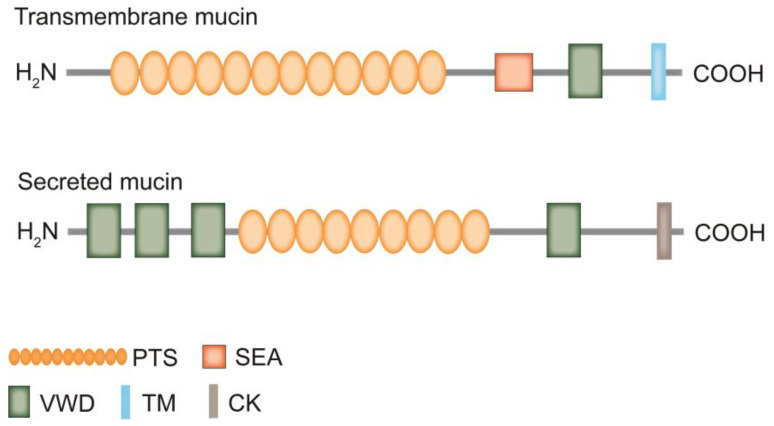
Schematic diagram of transmembrane mucin and secreted mucin structures. Both transmembrane mucins and secreted mucins have the C-terminal cytoplasmic tail, the N-terminal signal sequence, and the protein structural domain VWD. The central part of mucin is composed of multiple PTS domains, which contain tandem repeat sequences rich in Pro, Ser, and Thr. The size and number of tandem repeat sequences vary from mucin to mucin, which means that the size of mucins may vary considerably. These PTS structural domains undergo O-glycosylation to become mucin structural domains. In addition, transmembrane mucins have the transmembrane structural domain (TM) and no “CK” domain. Secreted mucins do not have the TM but consist of a large number of VWD domains, followed by a PTS domain and the “CK” domain.

Mucins are a class of high molecular weight glycoproteins (molecular weights typically ranging from 0.2 to 10 million Dalton) [1], which are mainly synthesized by goblet cells and combine with inorganic salts suspended in water to form mucus. They are widely expressed in the body and associated with many physiological and pathological processes [2]. Mucins cover the surfaces of the respiratory, digestive, gastrointestinal, and genitourinary tracts, protecting epithelial cells from infection, dehydration, and physical or chemical damage, providing protection and lubrication for the epithelial surface. So far, there are 20 kinds of mucin in the human body that have been discovered, including the secreted mucins MUC2, MUC5AC, MUC5B, MUC6, MUC19, MUC7, and MUC9 [3,4], as well as transmembrane mucins including MUC1, MUC3A, MUC3B, MUC4, MUC11, MUC12, MUC13, MUC15, MUC16, MUC17, MUC20, MUC21, and MUC22 [5]. In addition, some proteins belong to atypical or mucin-like protein molecules; atypical mucins including MUC10, MUC14, and MUC18. Mucin-like protein molecules exist in parasites, viruses, and fungi. For instance, *Herpes virus* has a mucin region, *Toxoplasma Gondii* has mucin-like structural domains, and *Candida albicans* has mucin-like proteins. Similar to the structure and function of mucins, mucin-like protein molecules have structural domains rich in proline (Pro), threonine(Thr), and serine (Ser), which can undergo extensive O-glycosylation and also act as barriers to protect cell membranes [6,7]. For example, the Msb2 glycoprotein of *Candida albicans* produces a mucosal layer to protect cells. This protein is considered as a functional analogue of mammalian MUC1/MUC2 [8]. Transmembrane mucins have a C-terminal cytoplasmic tail, a transmembrane region, and an extracellular portion, characterized by the sea urchin spermatoglycan, enterokinase and agrin structural domain (SEA) and the von Willebrand D (VWD). Unlike transmembrane mucins, secreted mucins lack transmembrane structural domains and exist in a secreted form [9]. Typical secreted mucins consist of a VWD domain rich in N-terminal cysteine, followed by a C-terminal “cystine knot” (“CK”) domain. The N-terminal participates in polymerization through intermolecular disulfide bonds, while the C-terminal “CK” domain participates in monomer dimerization [10] (Figure 1). Both types of mucins have a highly glycosylated protein core with multiple tandem repeats rich in Pro, Thr, or Ser, which is also known as the PTS domain. The size and number of tandem repeat sequences are significantly different in individual mucins, hence they are called variable number tandem repeats (VNTR) [1]. Secreted mucins are found to be responsible for conferring viscoelasticity to epithelial tissues, while transmembrane mucins are involved in maintaining epithelial cell polarity and cell signaling [11]. In addition, the expression patterns of mucin genes in the respiratory, digestive, and reproductive tracts are complex and strictly regulated. The expression of each mucin has organ and cell specificity [12]. Available studies have shown that the mucin components of the lungs are mainly MUC5AC and MUC5B. MUC5B is essential for cilia motility, while MUC5AC is more responsive to environmental or infectious factors, and elevated concentrations of MUC5AC may contribute to the development of chronic obstructive pulmonary disease (COPD) [13]. Terada found by immunohistochemistry that normal gastric mucosa specifically expresses MUC2, MUC5AC, and MUC6, but never MUC1, and colorectal crypt epithelial cells highly express MUC2, but never MUC1, MUC5AC, and MUC6 [14]. In the female reproductive tract, the major transmembrane mucins are MUC1 and MUC4, and the major secreted mucins are MUC5B and MUC5AC [15]. This evidence indicates that the expression of mucins is indeed organ and cell-specific.

**Figure 2:**
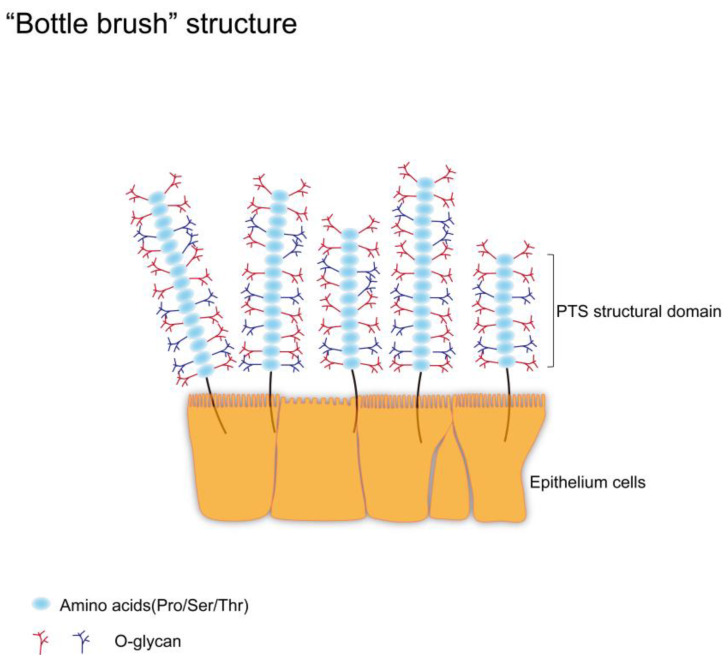
Schematic diagram of “bottle brush” structure. There are a large number of transmembrane mucins on epithelial cells. The PTS structural domains of these mucins are rich in Pro, Ser, and Thr residues, which are potential sites for O-glycosylation. When the PTS structural domain undergoes O-glycosylation, a densely arranged O-glycan scaffold is formed. These O-glycans account for 80% of the total mass of mucin and help maintain the high viscosity and rigid structure of mucin. The maintenance of this state is essential for mucins to fulfill their physiological roles.

As mentioned earlier, mucin possesses a highly glycosylated PTS domain. It is similar to a “bottle brush”, which allows carbohydrates to make up 80% of the total mass of the mucin. Since the PTS domain has a large and dense O-glycan chain structure, the O-glycans on mucins are collectively referred to as mucin glycans (Figure 2). Mucin glycans are beneficial for maintaining a high viscosity state. For example, in the stomach, high viscosity helps to lubricate indigestible lumen contents and accelerate gastric emptying. At the same time, they can protect epithelial cells from dehydration and mechanical forces during the passage of lumen contents [16]. However, the abnormally high mucin viscosity leads to an increased concentration of gallbladder bile and the possible formation of gallstones [17].

It should be noted that mucins encoded by the same gene may have different structures due to different glycosylation patterns. Meanwhile, their expression levels and glycan patterns may vary depending on the organization and species, which may lead to the occurrence of diseases [18]. This may be due to changes in the topology, function, and expression of individual glycosyltransferases and their molecular chaperones leading to glycan loss, which results in the production of aberrant glycan chains on mucins [19]. For example, the aberrant expression of several types of truncated O-glycans is mainly due to differences in the expression or activity of glycosyltransferases [20,21]. The most common truncated O-glycans include T antigen (Galβ1-3GalNAcα1-O-Ser/Thr), Tn antigen (GalNAcα1-O-Ser/Thr), and STn antigen (Neu5Acα2-6GalNAcα1-O-Ser/Thr), which are mainly caused by mutations in *COSMC*, the molecular chaperone encoding the molecule required for the formation of active C1GALT1 (core 1β1,3-galactosyltransferase) [22,23]. These abnormal glycosyltransferases and truncated O-glycans are involved in the occurrence and progression of cancer, and serve as important biomarkers for cancer diagnosis and treatment [24,25]. Existing studies have shown that alterations in intestinal mucin glycans are associated with a variety of intestinal disorders, and that they can lead to dysbiosis, which usually occurs in the early stages of enterocolitis [26]. Alterations in O-glycans on MUC2 have been observed in ulcerative colitis (UC), which refers to an increase in the number of truncated O-glycans Tn antigen, STn antigen, and a decrease in the number of complex O-glycans on MUC2. These abnormal mucin glycans will further aggravate UC [27]. In addition, during the development of cancer, the glycosylation pattern of mucins also changes. This abnormal glycosylation pattern can alter cell function and participate in cancer cell proliferation, invasion, metastasis, and angiogenesis [19]. For example, the mucins of normal breast epithelial cells contain a mixture of O-glycans, most of which are core 2 structure (GlcNAcβ1-6Galβ1-3GalNAcα1-O-Ser/Thr), but in breast cancer (BC), there is a decrease in core 2 structure and an increase in sialyl Lweis x (NeuAcα2-3Galβ1-4 [Fucα1-3] GlcNAc, SLe^x^) antigen, which can lead to enhanced adhesion of BC cells to endothelial cells [28].

## 2. Major Types of Glycosylation on Mucins

**Figure 3:**
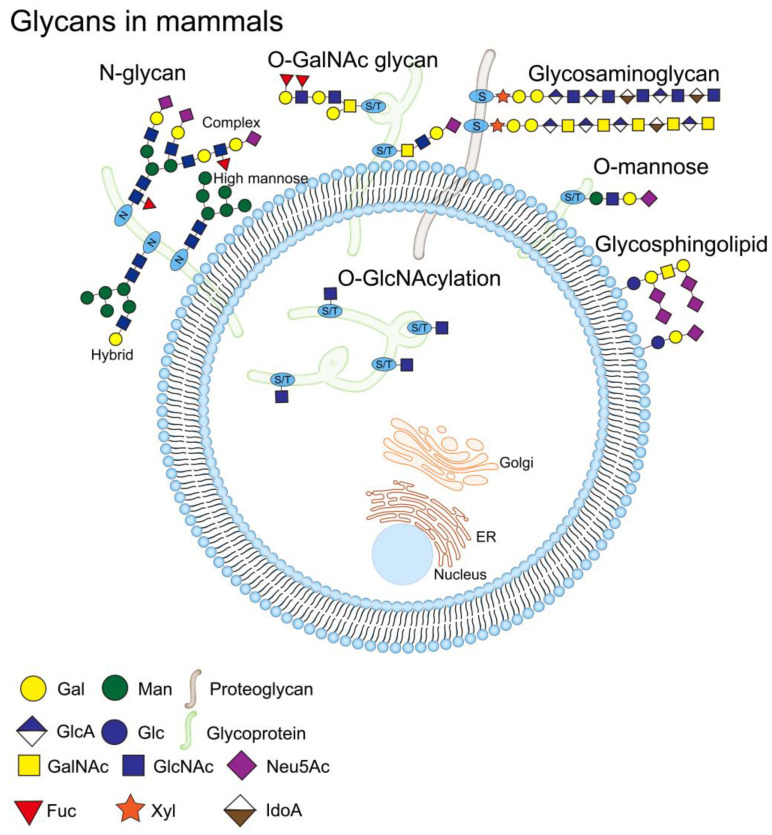
Schematic diagram of glycans in mammals. Glycosylation is one of the processes of the PTS; glycosylation occurs mainly in the ER and the Golgi, and there is a difference in the site of occurrence of each type of glycosylation. O-GalNAc glycosylation occurs mainly in the Golgi. N-glycosylation and GSLs are initiated in the ER and processed and terminated in the Golgi. O-fucosylation, O-glucosylation, O-galactosylation, O-N-acetylglucosamine (O-GlcNAc), O-mannosylation, and PGs all occur in the ER, and O-GlcNAcylation occurs in the cytoplasm. Mammalian cell surfaces are covered with a dense array of glycoproteins, including N-glycans (high mannose, hybrid, and complex N-glycan) and O-GalNAc glycans, etc. O-GlcNAcylation is present in cytoplasmic, nuclear, and mitochondrial glycoproteins. The glycan structure is represented according to the Symbolic Nomenclature for Glycans (SNFG) format [46].

The complexity of mucin structures is due to their long polypeptide chains and various post-translational modifications (PTMs), such as glycosylation, sulfation, and phosphorylation [11]. Among them, glycosylation is one of the main PTMs that define these mucins and affect their function, which mainly occurs in the endoplasmic reticulum (ER) and Golgi apparatus [29]. There are differences in the site of occurrence of each type of glycosylation; O-GalNAc glycosylation (also called mucin-type O-glycosylation) mainly occurs in the Golgi apparatus. N-glycosylation and glycosphingolipids (GSLs) are initiated in the ER and further processed and terminated in the Golgi apparatus. Other types of glycosylation, including O-fucosylation, O-glucosylation, O-galactosylation, O-N-acetylglucosamine (O-GlcNAc), O-mannosylation, and proteoglycans (PGs), occur in ER, while O-GlcNAcylation occurs in the cytoplasm, and their eventual localizations in the cells are also different [30,31,32,33,34] (Figure 3). Glycans are composed of ten monosaccharides: fucose (Fuc), galactose (Gal), glucose (Glc), N-acetylglucosamine (GlcNAc), N-acetylgalactosamine (GalNAc), glucuronic acid (GlcA), iduronic acid (IdoA), sialic acid (Sia), mannose (Man), and xylose (Xyl), which are linked by α or β bonding to form linear or branched structures [35,36,37]. Sia can provide a negative charge to the protein molecule, which introduces spatial repulsion in the side chains of the glycan chain. This in turn imparts rigid structural chains to the mucin, and these structures maximize the extension of the glycan chain [38]. Fuc can confer more neutral charge properties to mucins, which can maintain the high viscosity of mucins by regulating the interaction of water molecules (or other charged ions) with mucins [39].

The glycosylation on mucin is mainly O-GalNAc glycosylation (hereinafter referred to as O-glycosylation) and N-glycosylation [40]. These two types of glycosylation may occur simultaneously on mucin, depending on the amino acid residues on the mucin. N-glycosylation connects carbohydrates to the amide nitrogen of Asparagine (Asn), while O-glycosylation connects carbohydrates to the hydroxyl groups of Ser/Thr [41,42]. Compared to N-glycosylation, O-glycosylation is the main glycosylation modification on mucins [43]. The processes of N-glycosylation and O-glycosylation are interconnected, and it has been found that when N-glycosylation is blocked, O-glycosylation of sucrose isomaltase and intestinal dipeptidyl peptidase IV is also inhibited [44]. In addition, there are some shared carbohydrate antigens between N-glycosylation and O-glycosylation. For example, the typical tumor-associated antigens sialyl Lewis a (NeuAcα2-3Galβ1-3 [Fucα1-4] GlcNAc, SLe^a^) and SLe^x^, which can appear on both N-glycans and O-glycans [45].

### 2.1. O-Glycosylation

**Figure 4:**
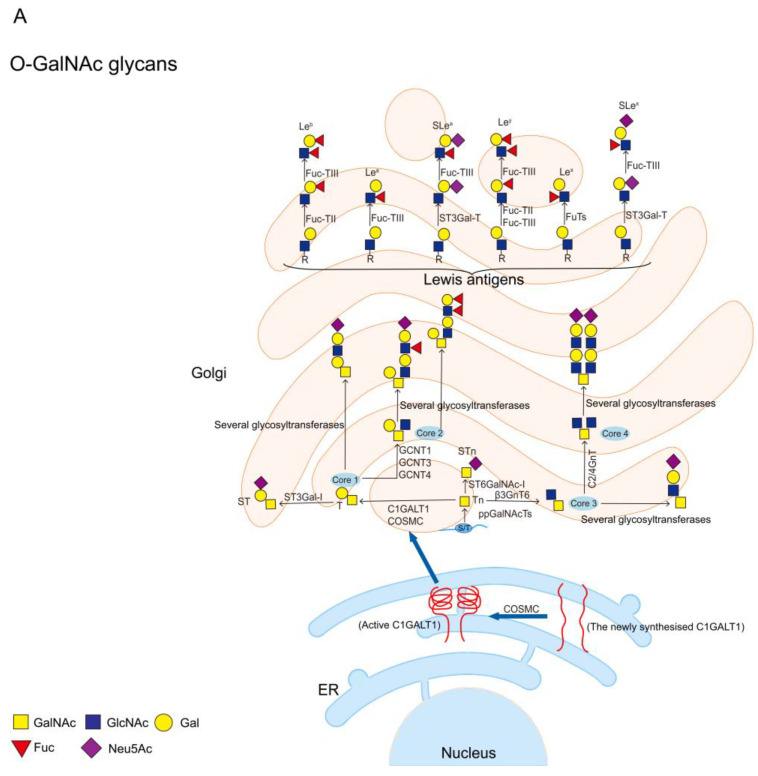

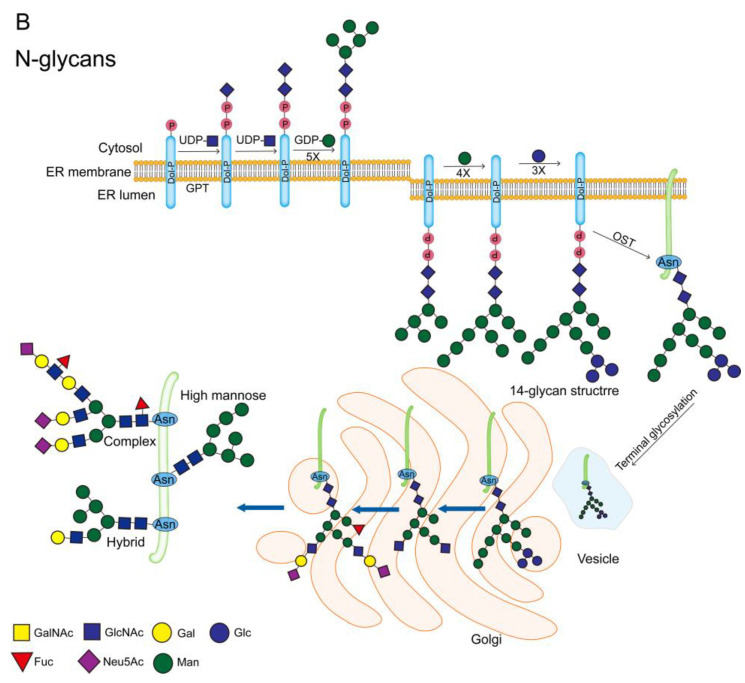
Schematic diagram of O-GalNAc glycans and N-glycans. (**A**) The first step in the synthesis of O-GalNAc glycans is the attachment of UDP-GalNAc to Ser/Thr residues of the protein in the presence of ppGalNAcTs to form the initial structure GalNAcα1-O-Ser/Thr (Tn antigen). Tn antigen is synthesized through the core 1 structure (T antigen) by C1GALT1. C1GALT1 needs to be rendered active by COSMC in the ER, and active C1GALT1 exists as a dimer. Tn antigens can also synthesize core 3 structures via β3GnT6, and core 1 and core 3 structures can be further synthesized into core 2 or core 4 structures. STn antigens are produced by the addition of sialic acid to the α1-6 linkage of the GalNAc residue of Tn via ST6GalNAc-I. These core structures are usually further extended and terminated with sialic and fucose residues. Finally, terminal Lewis antigens include Le^a^, Le^b^, Sle^a^, Le^x^, Le^y^, and Sle^x^, etc.; (**B**) N-glycosylation is initiated by transferring GlcNAc to Dol-P at the ER membrane to form Dol-P-P-GlcNAc, a process mediated by GPT. The 5 GDP-Man residues are then sequentially ligated on by specific glycosyltransferases to generate the Man_5_GlcNAc_2_-P-P-Dol structure. This structure is flipped into the ER lumen and the addition of 4 Man and 3 Glc continues to generate the 14-glycan structure, the precursor of the N-glycan. The 14-glycan structure is transferred by OST to the Asn residue of the protein. The 14-glycan structure is then vesicle-wrapped into the Golgi for terminal glycosylation, which involves the removal of mannose and the addition of other sugar groups. After terminal glycosylation, N-glycosylated proteins are divided into three main types, high mannose, hybrid, and complex. The glycan structure is represented according to the SNFG format [46].

More than 80% of the glycan chains on mucin are O-glycans. The first step of O-glycosylation is to add GalNAc from UDP-GalNAc to the Ser/Thr residue of the mucin PTS domain under the action of the peptide transferases family (ppGalNAcTs) to form the initial structure Tn antigen. There are 19 ppGalNAcTs family members in mice, 20 in mammals, and 10 in drosophila, and the expression of these enzymes is substrate and tissue-specific [10]. Downward extension of the Tn antigen can produce eight different core structures, with core 1–4 being the four main core structures that are widely expressed in vivo. Expression of other core structures 5–8 is very limited, and expression of core 7 is not found in humans [41]. In detail, Tn antigen is synthesized into core 1 structure (also called T antigen, by the action of C1GALT1). In mammals, C1GALT1 requires the action of the molecular chaperone COSMC to fulfill its normal biological role [47]. COSMC is an ER-localized chaperone that interacts with newly synthesized C1GALT1 to promote the correct folding of C1GALT1 and keep C1GALT1 active [48]. The Tn antigen can also be synthesized by core 3 β1,3-N-acetylglucosaminyl transferase (β3GnT6) to form a core 3 structure (GlcNAcβ1-3GalNAcα1-O-Ser/Thr), or by sialyl transferase ST6GalNAc-I to synthesise STn antigen. T antigen and core 3 structures can be further branched to core 2 or core 4 structures by adding other sugar groups, and the four core structures are usually further extended and covered by terminal residues [49]. These residues include Fuc (Fucα1-2, -3 or -4) and Sia (NeuAc(Gc)α2-3 or -6) [9] (Figure 4A). The most common structure on mucins is the sialylated core 1 structure (also called ST antigen, NeuAcα2-3Galβ1-3 [NeuAcα2-6]+/-GalNAcα1-O-Ser/Thr), as well as the sialylated core 2 structure (NeuAcα2-3Galβ1-3[NeuAcα2-3Galβ1-3/4GlcNAcβ1-6] GalNAcα1-O-Ser/Thr) [41].

Normal initiation and extension of O-glycosylation on mucins affects a variety of biological processes, including cellular aspects (targeted transport of glycoproteins), molecular aspects (protein conformation, protein hydrolysis resistance), and aspects of cellular communication (cell-cell and cell-substrate interactions) [50]. On the one hand, O-glycans on mucins can inhibit the virulence of pathogens; for example, O-glycans on the salivary mucin MUC5B can inhibit the virulence of the oral pathogen *Streptococcus mutans* [51]. On the other hand, O-glycans on mucins are essential for maintaining the normal state of the eyes. O-glycans on mucins are also involved in maintaining the highly extended and rigid structure of mucins, which enables mucin and its O-glycan to maintain the sugar calyx structure at the top of the eye surface [52]. Moreover, O-glycans on mucin can interact with galactose lectins; for example, O-glycans on MUC16 and MUC1 interact with galactose lectins -1 (Gal-1) and -3 (Gal-3) to promote the formation of protective lattices in the cornea [53,54]. Abnormal O-glycosylation of mucins also leads to congenital disorders of glycosylation (CDG). So far, most CDGs are caused by defects in the coding region or spliceosomes of glycosyltransferases, which can cause abnormal truncated structures (Tn, STn, T, ST) or extended structures (SLe^x^, SLe^a^) on glycan chains [55,56].

Finally, normal biosynthesis of O-glycans on mucins is essential for cancer development. Taking MUC1 as an example, it is clinically called carbohydrate antigen 153 (CA153). As early as May 1996, the American Society of Clinical Oncology (ASCO) evaluated MUC1 and designated it as a tumor marker for BC [57]. MUC1 expression and its aberrant O-glycosylation are frequently observed in epithelial cancers, and this aberrant O-glycosylation mainly refers to the increased expression of truncated O-glycans as well as aberrant extended structures, which play a key role in the occurrence, progression, and metastasis of cancer [58]. Expression of aberrant ST structures on MUC1 can promote cancer cell growth, and the immune system seems to play a role in this [59]. Existing studies have shown that the loss of O-glycans derived from core 1 can lead to dysregulation of MUC1 expression, damage to the gastric mucus layer, and altered gastric acid balance, leading to the occurrence of gastritis and gastric cancer (GC) [60]. Subsequently, studies found that MUC1 can promote GC metastasis, is associated with poor prognosis, and appears to be involved in the progression of diffuse gastric cancer (DGC) [61,62].

### 2.2. N-Glycosylation

As mentioned above, the biological properties of mucins are associated with O-glycosylation. In turn, their N-glycosylation is important in the processing and biological properties of mucins [44]. N-glycosylation is initiated by the transfer of GlcNAc from UDP-GlcNAc to Dol-P lipid molecules on the ER membrane to form Dol-P-P-GlcNAc; this process is mediated by UDP-N-acetylglucosamine-dolichyl-phosphate N-acetylglucosaminephosphotransferase (DAPGT1/GPT). Then, UDP-GlcNAc and 5 GDP-Man residues are sequentially linked by specific glycosyltransferases to generate the Man_5_GlcNAc_2_-P-P-Dol structure. This structure flips into the ER lumen and proceeds to add 4 Man and 3 Glc to produce the Glc_3_Man_9_GlcNAc_2_-P-P-Dol structure (14-glycan structure), the precursor of N-glycans. The 14-glycan structure is transferred by oligosaccharyl transferase (OST) to asparagine residues in the N-glycosylation site (Asn-X-Ser/Thr) of the nascent protein [42]. After that, the 14-glycan structure is encapsulated by vesicles and enters the Golgi for terminal glycosylation, which involves removing mannose and adding other sugar groups. After terminal glycosylation, N-glycosylated proteins are divided into three main types, high mannose, hybrid, and complex N-glycan [63] (Figure 4B).

N-glycosylation is important for the dimerization and multimerization of mucins, which can provide stability to mucins by promoting folding and inhibiting degradation and is essential for mucin folding, sorting, and secretion [44,64]. The presence of N-glycans at N9 site on MUC2 helps to maintain the folding rate of MUC2 monomers, which is slow enough to allow for the structural maturity and stability of MUC2 dimers. This means that the formation of MUC2 dimers cannot be separated from the N-glycosylation of MUC2 [65]. Asker et al. found that inhibition of N-glycosylation of MUC2 with tunicamycin resulted in a reduction of dimers and oligomers of MUC2 [66]. In addition, mucins and their N-glycans are also important for maintaining the normal state of the eyes. Taniguchi et al. found that the expression of MUC16 was downregulated and the function of the glycocalyx barrier was disrupted after inhibiting N-glycosylation in human corneal epithelial cells with tunicamycin. Notably, N-glycans in human corneal epithelial cells are mainly complex structures, which are galactose lectin ligands. N-glycans on MUC16 can promote the binding of MUC16 and Gal-3. Compared to O-glycans, Gal-3 has a higher affinity for N-glycans, indicating that complex N-glycans are the preferred ligand for galactose lectins [64].

Abnormal N-glycosylation of mucins is also a mark of cancer [24]. Expression of highly branched β1-6 N-linked glycans is significantly upregulated in various cancer cells compared to normal cells, which is associated with cancer cell proliferation, invasion, and metastasis [67]. N-glycans can promote the binding of MUC16 and glycosylphosphatidylinositol anchored glycoprotein mesothelin, thereby promoting peritoneal metastasis of ovarian cancer (OV) [68]. Although N-glycosylation on mucins has been less studied in carcinogenesis, the role of N-glycosylation on other proteins in carcinogenesis has been widely reported. N-glycosylation of Asn294 and Asn454 of Mer tyrosine kinase (MerTK) promotes the proliferation of hepatocellular carcinoma (HCC) by stabilizing the expression of MerTK [69]. N-glycosylation at the Asn157 site of CD82 regulates colon cancer (COAD) transfer and adhesion by inhibiting epithelial–mesenchymal transition (EMT) [70].

### 2.3. PGs and GSLs

Finally, we elaborate on PGs and GSLs. Although they are not the main glycosylation types on mucins, their role in cancer occurrence and progression cannot be ignored. PGs are an important component of glycocalyx, consisting of protein core and glycosaminoglycans (GAGs) [71]. The GAGs family is classified according to its chemical composition, including chondroitin sulfate (CS), dermin sulfate (DS), keratin sulfate (KS), heparan sulfate (HS), and hyaluronic acid (HA) [72,73]. Some PGs (such as syndecans) can be simultaneously modified by different GAGs, which can carry CS, DS, and HS. In summary, PGs are a highly heterogeneous group of proteins that differ through alterations in the structure of the protein core as well as differences in GAGs type and length [71]. As important molecules in cellular signal transduction processes, proteoglycans have great significance in cancer cell proliferation and metastasis [74]. PGs can regulate cancer progression by binding to growth factors, adhesion factors, and cytokines [75,76]. For example, Syndecan 4 promotes cancer cell proliferation and migration by binding to fibronectin to activate the MAPK signaling pathway [77]. Furthermore, as an important component of the extracellular matrix (ECM), PGs can interact with cell proliferation-related signaling pathways (including NF-κB and EGFR signaling pathways) to regulate cancer cell proliferation [76]. The small Leucine (Leu) rich PG families (SLRPGs) are upregulated in expression in various cancers [78]. Lumican is an SLRPG, and studies have shown that Lumican regulates cancer cell proliferation, invasion, and metastasis through different mechanisms [79]. For example, lumican promotes gastric cancer cell growth by activating the integrin β1/FAK signaling pathway. Lumican is upregulated in HCC. Knockdown of Lumican inhibited invasion and migration of HCC [80].

GSLs consist of three basic components including sphingosine, fatty acids, and carbohydrate residues [81]. They have important biological functions, including regulation of cellular signal transduction and mediation of cell recognition and adhesion [82]. GSLs are upregulated in cancer cells, some of which have been proven to be adhesion molecules involved in cancer cell metastasis which can regulate cancer cell signal transduction [83]. Many GSLs are useful biomarkers or targets for cancer diagnosis or treatment [82]. For example, ganglioside GM3 is a simple structured GSL. It can inhibit the proliferation and cell adhesion of bladder cancer (BLCA) cells [84]. α-Galactosylceramide (α-GalCer) is a type of GSLs, which can exert anticancer effects by upregulating IFN-γ expression [85]. In addition, treatment with α-GalCer significantly inhibited the growth of colon cancer (COAD) in vivo.

## 3. The Mechanisms of Abnormal Mucin Glycans Production

As mentioned earlier, normal glycosylation of mucin is particularly important for the maintenance of body health. When abnormal glycosylation occurs, it not only affects the expression of mucins, but also affects their biological functions. Several main factors contribute to the occurrence of abnormal glycosylation on mucins, including the alterations in the activity and localization of glycosyltransferases, alterations in the pH of Golgi, and the efficiency of nucleotide transport proteins [86]. Each of these mechanisms will be described in turn below.

### 3.1. The Activities and Localizations of Glycosyltransferases

The activity of glycosyltransferase and its localization in the Golgi apparatus are the basis for normal glycosylation synthesis, which determines the structure and quantity of O-glycans [19]. Abnormal expression and activity of glycosyltransferases have been shown to be a factor in cancer development [55]. For example, N-Acetylgalactosaminyltransferase 7 (GALNT7) is upregulated in prostate cancer (PRAD) and can promote its proliferation by modifying the O-glycosylation of PRAD cells [87]. Notably, O-glycosyltransferase activity can depend on N-glycosylation. Glycosyltransferases lacking N-glycosylation may misfold and aggregate in the ER. Prorok Hamon et al. found that fucosyltransferase 7 (FUT7) has two N-glycosylation sites at Asn81 and Asn291, and changes in N-glycosylation at both sites can significantly decrease FUT7 activity [88]. Subsequently, Ruggiero et al. reported that ST3Gal-II has two N-glycosylation sites at Asn92 and Asn211. When N-glycosylation is absent at Asn211 site, ST3Gal-II undergoes misfolding, stalls in the ER, and loses activity [89]. These abnormal glycosyltransferases further affect the biological process of glycosylation.

The localization of glycosyltransferase on the Golgi apparatus is an important factor in controlling glycan biosynthesis. The enzymes that synthesize core 1 and core 2 are mainly located in the cis Golgi apparatus, while the enzymes that synthesize terminal structures are mainly located in the trans Golgi apparatus [19]. The activity of the glycosyltransferase also changes when the glycosyltransferase is transferred from the cis Golgi to trans Golgi. Researchers transferred core 2 β-1,6-acetylglucosaminyltransferase (C2GnT, mainly located at cis Golgi apparatus) to trans Golgi apparatus and analyzed it. The results showed that the expression of core 2 branched oligosaccharides was drastically reduced after the transfer of C2GnT to the trans Golgi apparatus [90]. On the other hand, if the first glycosyl (GalNAc) is added later than the interval where the elongase is located, then GalNAc cannot be used as a glycosylation substrate [91].

### 3.2. Changes in Golgi pH

It is well known that the acidic pH of the Golgi lumen is essential for the correct glycosylation, transport, and sorting of proteins and lipids in the organelle [92]. Elevated Golgi pH appears to be directly related to the expression of T antigens in cancer cells, and it may also affect the localization and distribution of glycosyltransferases in the Golgi apparatus [93]. Axelsson et al. stimulated HeLa and LS174T cells with ammonium chloride (NH_4_Cl) or proton pump inhibitor (BafA1) and found that they effectively neutralized pH, which resulted in the relocation of N-Acetylgalactosaminyltransferase 1 (GALNT1) and N-Acetylgalactosaminyltransferase (GALNT2) from the ER to the Golgi apparatus [94]. Changes in Golgi pH can lead to incorrect synthesis of O-glycans and N-glycans [93]. There is evidence that stimulation of fibroblast COS-7 with pH gradient dissipating drugs (BafA1, chloroquine, or NH_4_Cl) leads to an increase in Golgi pH and upregulation of intracellular T antigen expression [91]. Treating colorectal cancer (CRC) cell line LS174T with BafA1 promotes the sulfation of mucin and upregulates the expression of T antigen [95].

### 3.3. Efficiency of Nucleotide Transporters

Before a glycosylation reaction can occur in eukaryotes, the activated sugar group must be translocated to the Golgi or ER, and only there can the sugar group be used as a substrate by glycosyltransferases. This is a task performed by a family of nucleotide sugar transporter proteins (NSTs) [96]. Mutations in NSTs or inefficient transport are associated with impaired glycosylation. For example, sialic acid is transported into the Golgi as CMP-sialic acid (CMP-Sia), which is carried out by the CMP-Sia transporter protein (CST). CST mutations can lead to incomplete sialylation and thus abnormal glycosylation [97]. CST can transport CMP-Sia and UDP-Galactose (UDP-Gal). Mutations at the Tyr214 and Ser216 sites on CST can lead to a loss of its transport activity towards CMP-Sia, but do not affect its transport activity towards UDP-Gal. That is, Tyr214 and Ser216 on CST are essential for the CMP-Sia transporter [98].

## 4. The Role of Abnormal Mucin Glycans in Cancer Development

Glycosylation is a highly dynamic process that produces significant changes during cancer development. Compared to normal cells, cancer cells express more branched N-glycans, higher levels of fucose, sialic acid glycans, and truncated O-glycans. This aberrant glycosylation is now widely regarded as one of the unique marks of cancer [24]. Under physiological conditions, mucin glycans are involved in the composition of the mucus barrier, they are the first responders of epithelial cells to mechanical or chemical damage, helping to maintain environmental balance within the body [99]. However, their role in protecting and repairing epithelial cells turns into a sharp edge in the carcinogenesis process. Abnormal mucin glycans are involved in the occurrence and development of cancer, and their role in cancer cell proliferation, adhesion, and invasion has been fully demonstrated. Here, we focus on the most studied MUC1 as an example. The expression of MUC1 is generally upregulated in most cancer cells, and the upregulated MUC1 is mainly in the plasma membrane and cytoplasm of cancer cells [100]. In addition to elevated expression, MUC1 on cancer cells undergoes aberrant glycosylation [101]. Below we will summarize the mechanisms by which aberrant mucin glycans contribute to cancer development.

### 4.1. Proliferative Capacity

MUC1 on normal breast epithelial cells mainly carries the core 2 structure. During the development of BC, core 2 structure on MUC1 is absent and core 1 structure is increased. To determine whether this change will promote the proliferation of BC cells in vivo, Mungul et al. constructed MUC1 cell lines expressing core 1 and core 2, respectively. The results showed that cells expressing core 1 structure proliferated faster than cells expressing core 2 structure [102]. Moreover, it has been well established that C1GALT1/N-Acetylgalactosaminyltransferase 6 (GALNT6) can regulate the O-glycan structure on MUC1 and activate the MUC1-C/β-catenin signaling pathway to promote the proliferation of BC cell lines [103,104]. On the other hand, in PRAD, upregulation of the expression of glucosaminyl (N-acetyl) transferase 1 (GCNT1) leads to aberrant expression of SLe^x^ on MUC1, but whether this aberrant O-glycosylation is involved in prostate carcinogenesis has not been clearly stated [105]. Researchers have characterized MUC1 with O-glycans, and subsequently found that in prostate cancer, the O-glycans on MUC1 are mainly core 2 structure, followed by fucosylated types of core 2 structure. Overexpression of MUC1 did not affect the proliferation of PRAD cells, but may promote PRAD metastasis. This suggests that the pro-carcinogenic mechanism of MUC1 in prostate cancer may be different from other epithelial cancers such as BC, COAD, and pancreatic cancer (PAAD) [106].

### 4.2. Loss of Adhesion

**Figure 5:**
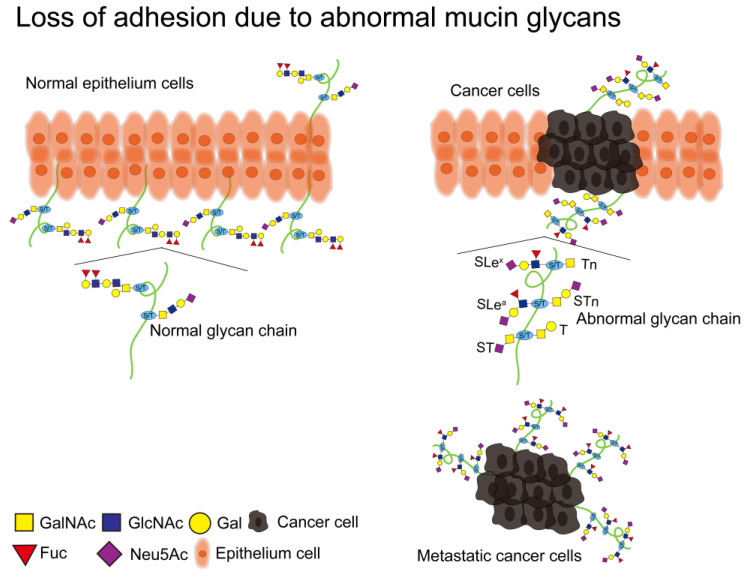
Glycans in mammals. Normal mucins have intact glycan chains on them that are involved in maintaining the morphology, structure, and function of epithelial cells. Abnormal alterations in glycosylation on mucins may lead to cellular carcinogenesis. A large number of truncated O-glycans (Tn, T, ST, STn) and extended O-glycans with core 2 structures (SLe^a^, SLe^x^) are present on cancer cells. Among them, the SLe^a^ and SLe^x^ structures lead to the detachment of cancer cells from the primary lesion and metastasis. The glycan structure is represented according to the SNFG format [46].

Cell adhesion molecules (CAMs) are a class of proteins located on the surface of cells that mediate cell-to-cell or cell-to-ECM contact and binding. As a form of information exchange between cells, they can undergo adhesion by recognizing specific adhesion receptors [107]. CAMs consist of four main families (integrins, cadherins, immunoglobulins, and selectins). Downregulation of cadherin, and upregulation of integrins, immunoglobulins, and selectins leads to loss of adhesion of cancer cells from the primary site, thus promoting metastasis of cancer cells [108]. The O-glycan structure on mucins is one of the most important factors in cell adhesion or anti-adhesion, and it is mainly changed in SLe^a^ and SLe^x^, leading to loss of adhesion in cancer cells [11] (Figure 5). SLe^a^ and SLe^x^ on MUC1 cover CAMs on the surface of cancer cells, thus causing cancer cells to lose adhesion, which has been confirmed in different cancers [109]. Rodriguez et al. found that MUC1 on COAD cells carries SLe^a^ and SLe^x^, and that SLe^a^ and SLe^x^ modify MUC1 into an epitope of E-selectin, which may be one of the molecular mechanisms by which MUC1 promotes COAD metastasis [110]. Solatycka et al. found that overexpression of MUC1 in BC cells resulted in upregulation of T antigen expression and deletion of SLe^x^ expression, and overexpression of MUC1 altered the adhesion ability of breast cancer cells [111]. Park et al. found that overexpression of MUC1 in breast cancer cells induced abnormalities in CAMs (β-catenin and E-cadherin), resulting in an anti-adhesion effect [112]. In cigarette smoke-induced pneumonia, abnormal glycosylation on MUC1 can promote E-cadherin degradation, thereby promoting EMT [113].

### 4.3. Cancer Metastasis

Most cancer patient deaths result from cancer metastasis, and clinically upregulated SLe^x^ expression is usually associated with poor prognosis in cancer patients [114]. In addition, sialic acid glycans on MUC1 can bind to lectins to promote the adhesion of cancer cells to vascular endothelial cells or promote the production of the tumor microenvironment, which is conducive to cancer progression and metastasis [24,115]. Cell surface lectins that can bind to MUC1 are divided into three major classes (C-type, S-type, and I-type lectins) [58]. The interaction of MUC1 with lectins is enhanced when there is an abnormal increase in SLe^a^ and SLe^x^ on MUC1, which promotes the aggregation of cancer cells and their metastasis to distal sites [58,116]. When lectin (Siglec-9) binds to the ST structure on MUC1 of myeloma cells, it promotes the formation of the tumor microenvironment (TME) and thus metastasis [59,117]. In addition, MUC1 is also a natural ligand for Gal-3, and the interaction of Gal-3 with MUC1 promotes the adhesion of already disseminated cancer cells to the endothelium of blood vessels, thus metastasis [118]. Finally, a possible mechanism by which MUC1 promotes cancer cell metastasis is related to anoikis. Piyush et al. proposed in 2017 that truncated O-glycans on MUC1 can enhance the anti-anoikis properties of cancer cells, leading to loss of cancer cell adhesion and metastasis [119].

### 4.4. Cancer Immune Escape

Tumor immune escape is essential to ensure the survival of cancer cells, which is defined as the escape of cancer cells from recognition and attack by the immune system through various mechanisms. Tumor immune escape is induced by several factors, including loss or alteration of cancer antigenic substances, weakening of cancer immunogenicity, epigenetic changes in cancer cells, changes in intracellular signaling pathways in cancer cells, etc. [120]. A large amount of evidence suggests that upregulated mucins in most adenocarcinoma cells are the main shielding medium for cancer cells to evade immune system surveillance, and abnormal glycosylation of mucins can help cancer cells evade immune system surveillance. This is mostly mediated by lectins, which affect the recognition of tumor antigens by the immune system by binding to aberrant mucin glycans [121].

SLe^a^ and SLe^x^ on MUC1 not only play a role in cancer cell adhesion and metastasis, but also compete with selectin ligands on leukocytes, interfering with their recognition and clearance of cancer cells, thereby helping cancer cells escape immune system surveillance [109]. Furthermore, when lectins are recruited to highly sialylated cancer cells, they prevent Natural Killer (NK) cells from recognizing cancer cells and block their initial immune response to cancer cells [58]. Overaccumulation of Tn and STn on MUC1 is particularly important for tumor cell immune escape. Moreover, aberrant glycans on mucins modulate the susceptibility to NK cell-mediated antibody-dependent cytotoxicity (ADCC) and cytotoxic T lymphocyte (CTL)-mediated killing [122].

### 4.5. Cancer Chemoresistance

Currently, targeting mucins in cancer cells is challenging due to the resistance of cancer cells to chemotherapeutic drugs [123]. The mucin barrier is one of the causes of chemoresistance in cancer cells [124]. Most mucins are negatively charged and can interact electrostatically with positively charged drugs, thereby inhibiting drug diffusion in the body. Effective small molecule inhibitors can disrupt mucin synthesis and help drugs reach their targets to overcome chemoresistance [123]. Rao et al. found that mucin synthesis could be blocked using glucosaminyl (N-acetyl) transferase 3 (GCNT3) inhibitor, which could be used alone or in combination with gemcitabine to inhibit cancer cell growth [123].

In addition, there is a direct relationship between chemical resistance and the expression of abnormal glycans in cancer cells [125]. Inhibition of N-glycosylation can affect the expression of EGFR and IGF1R, and enhance the sensitivity of PAAD cells to chemotherapy drugs [126]. Different glycosyltransferases may regulate the resistance of chemotherapy drugs [125]. Silencing GALNT6 not only damages the expression of glycans, but also makes drug-resistant cells more sensitive to anticancer drugs [125]. Jin et al. also confirmed that the expression of N-Acetylgalactosaminyltransferase 14 (GALNT14) is related to the multidrug resistance (MDR) of BC cells, and knocking down GALNT14 will make BC cells sensitive to adriamycin [127]. Finally, truncated O-glycans are a common tumor-related carbohydrate that can help cancer cells resist chemotherapy drugs [128,129].

### 4.6. Carcinogenic Pathogens

As of 2018, the four most important of the 11 infectious agents defined by the International Agency for Research on Cancer (IARC) as Class I carcinogens are *Helicobacter pylori* (*Hp*), high-risk human papillomavirus (HPV), hepatitis B virus (HBV), and hepatitis C virus (HCV) [130]. Abnormal mucin glycans may be either favourable or unfavourable to pathogens infecting the body, as they can affect the resistance of the host [131,132]. It has been shown that abnormal mucin glycans can provide a favourable environment for pathogens in the mucus layer during pathogen infection, thus increasing the survival of pathogens [133].

Infection with *Hp* is the greatest risk factor for GC [134]. It was shown that knockout of fucosyltransferase 2 (Fut2) resulted in downregulation of the expression of α1,2-fucosylated structures and upregulation of SLe^a^ expression on MUC5AC. This aberrant fucosylation on MUC5AC would help *Hp* to colonize the gastric mucosa [135]. In addition, Skoog et al. analyzed the O-glycans on gastric mucin infected and uninfected with *Hp*, respectively. It was found that after infection with *Hp*, the O-glycan structure on gastric mucin became more numerous and complex [136]. HPV can lead to the occurrence of cervical cancer (CESC). Solórzano et al. conducted O-glycan analysis of mucins in CESC infected with HPV, and the results showed that HPV infection can cause abnormal fucosylation of mucins, which can affect the invasion of CESC cells [137]. Ahmad et al. found that during HCV/HBV infection, the binding of O-glycans at the Ser204 site of insulin-like growth factor to cellular growth factor is reduced, which can lead to the occurrence of HCC [138].

Speaking of oncogenic pathogens here, it is important to mention Epstein-Barr virus (EBV), which is the first human virus directly associated with cancer and has been linked to the pathogenesis of Burkitt’s lymphoma (BL), Hodgkin’s lymphoma (HL), non-Hodgkin’s lymphoma (NHL), and nasopharyngeal carcinoma (NPC). Not only that, it is also associated with epithelial malignancies such as GC and BC [139]. EBV can upregulate the expression of GCNT3 by affecting the NF-κB signaling pathway, which promotes the proliferation and migration of GC cells [140]. In addition, EBV latent membrane protein 1 (LMP1) can activate STAT signaling by regulating MUC1 expression, which ultimately promotes cancer invasion and metastasis [141]. Unfortunately, relevant studies are currently limited to interactions between EBV and aberrant glycosylation or mucin expression, whether EBV can interact with glycosylation on mucins is currently unknown. In summary, it is worth considering whether EBV can cause cancer by regulating glycosylation on mucins.

## 5. MUC1-Based Cancer Diagnosis and Targeted Therapy

Aberrant glycosylation may induce a range of different cancer-associated epitopes. These epitopes can be divided into three categories, including truncated O-glycans (T, Tn, and STn antigens), terminal structures such as SLe^a^ and SLe^x^ antigens in glycoproteins, and lipids [56,142,143]. Abnormal expression of mucins is often observed during the development of cancer [143]. Therefore, using abnormal mucin glycans on cancer cells as an immunotherapy strategy can provide a basis for the diagnosis and treatment of cancer [144]. Here, some clinical treatment strategies based on mucin-targeted cancers are summarized and listed in Table 1.

MUC1 was initially detected by monoclonal antibodies (mAbs) targeting human milk fat globules (HMFG) [160]. It is the first cloned mucin and the best-characterized mucin to date [161,162]. The N-terminal structural domain of MUC1 has a VNTR sequence on which glycosylation occurs, and each VNTR consists of 20 amino acids (GVTSAPDTRPAPGSTAPPAH). The PDTR sequence is the core of MUC1, and is also defined as the unique extracellular domain of MUC1 [163]. The PDTRP sequence can be recognized by mAbs and appears to be the immunodominant epitope [164,165]. MUC1 has always been considered a target for cancer therapy and diagnosis because it is upregulated and frequently aberrantly glycosylated in most adenocarcinomas [162]. Recently, in a ranking of 75 tumor antigens based on characteristics such as therapeutic function, immunogenicity, oncogenicity, and specificity, MUC1 received the second-best rating (after WT1), underlining its potential for medical research and vaccine development [166]. Here, we describe the antibodies, radiopharmaceuticals, vaccines, and CAR-T cell therapies based on MUC1 and summarize them in a table (Table 2).

### 5.1. Antibodies

Antibodies against aberrant glycosylation on MUC1 (MUC1-Tn, MUC1-STn) have been demonstrated in BC, OV, and PRAD [167]. As early as 1987, Burchell et al. designed an mAb (SM3) against the PDTRP sequence on MUC1, which responded to 91% of BC, but showed little response to benign breast tumors, normal resting, pregnant or breastfeeding mammary glands [168,169]. Subsequently, Rokhlin et al. in 1998 designed mAb (5E10) targeting the extracellular structural domain of MUC1 [170]. The specificity of 5E10 is relatively good. It can react with benign and malignant PRAD, but has no reactivity to normal prostate tissue [170]. In addition, humanized antibody (5E5) directed against the MUC1-Tn/STn epitope specifically recognizes the GSTAP sequence of MUC1 and its immunoreactivity against MUC1-Tn is better; it can activate the NK cell-mediated antibody-dependent cellular cytotoxicity (ADCC) process in vitro [171,172,173]. To this day, various antibodies have been created against different structural domains of MUC1, and they have been clinically successful. However, antibodies against the VNTR epitope have not yet yielded results in clinical trials, which have failed mainly due to the shedding of the VNTR epitope and its release into the circulation, thus preventing the binding of antibodies to the structural domain of MUC1 on the surface of cancer cells [174].

Therefore, in recent years, most attention has been focused on designing antibodies or antibody-conjugated drugs targeting other domains of MUC1. Recently, an mAb (TAB004) specifically targeting the extracellular structural domain of human MUC1 has been developed. It is reported that upon treatment of PAAD cells with TAB004, the binding of TAB004 to MUC1 induced ER stress and anoikis in the PAAD cells, which resulted in an inhibition of the proliferative capacity of the PAAD cells. When TAB004 was combined with 5-FU, it enhanced the survival of PAAD mice [175]. Wu et al. developed an mAb targeting MUC1-C (hMUC1), which recognized recombinant MUC1 as well as natural MUC1-C in BC cells, and hMUC1 significantly inhibited BC cell proliferation in vivo [176]. Subsequently, they developed a humanized MUC1 antibody (HzMUC1) targeting the interaction region between MUC1-N and MUC1-C. They coupled HzMUC1 with monomethyloristatin (MMAE) to generate an antibody-drug coupling (ADC), which can inhibit the growth of PAAD cells by inducing PAAD cell cycle arrest and apoptosis [177].

### 5.2. Radiopharmaceuticals

Despite the high specificity and affinity of mAbs, they have some drawbacks such as high immunogenicity, limited penetration into tumor tissue, large size, and long plasma half-life. An effective strategy to reduce these drawbacks is to use radioactive elements to label mAbs, which is also known as radiopharmaceuticals [178]. Radiopharmaceuticals consist of two parts, namely radioactive elements and carriers. The carrier is a class of biologically active molecules, typically antibodies, peptides, and aptamers [179].

The specific antibody (PR81) of MUC1 has a high affinity for BC, so Salouti et al. generated ^99m^Tc-HYNIC-PR81 by radiolabeling PR81 with ^99m^Tc, which improved the stability and immune reactivity of PR81 in vitro [180]. In addition, the anti-MUC1 aptamer was labeled with ^99m^Tc to generate a labeled drug delivery system (DDS), which was subsequently used for in vivo imaging of triple-negative breast cancer (TNBC). The results showed that the DDS had a high tumor uptake (5%) and great in vivo imaging properties [181]. Jammaz et al. radiolabeled the MUC1-FA-SFB hybrid conjugate with ^18^F, and the labeled conjugate (MUC1-FA—[^18^F] SFB) has high affinity and specificity for BC. In addition, MUC1-FA—[^18^F] SFB may be a PET imaging probe for BC detection and prognosis monitoring [182]. Therefore, there is great potential for labeling antibodies or aptamers to MUC1 with radionuclides, which could enhance the therapeutic effect of drugs for cancer treatment [183]. Compared to traditional treatment methods, radiopharmaceutical therapy (RPT) is the least toxic and most targeted form of effective cancer treatment [184,185]. Although RPT has been shown to be an effective cancer treatment and has been clinically studied for over 40 years, it has not become part of the cancer treatment armamentarium like other therapies [184]. One possible reason is that the targeted pathways of these drugs are not involved in the formation of the malignant phenotype of cancer [186].

Currently, drugs for cancer therapy have shortcomings such as high toxicity and systemic side effects due to the non-specific distribution of the drugs. Nanomedicine may improve this disadvantage by adjusting the delivery system [187]. The coupling of mAbs with nanoparticles (NPs) generates antibody nanocouplings (Ab-NPs), which significantly improve therapeutic specificity [188]. Chemokine receptor CXCR4 antagonist (PCX) can inhibit the migration of cholangiocarcinoma (CCA) cells. The coupling of PCX with anti-miR-210 NPs significantly inhibits the proliferation of CCA cells and improves the sensitivity of CCA cells to chemotherapy drugs [189]. Nevertheless, these traditional NPs still have some drawbacks. For example, they cannot be visualized during drug delivery. To visualize NPs, Liu et al. constructed a MUC1-based dual-targeting DNA tetrahedral NPs (MUC1-Td-AS1411) for BC cell imaging and targeted drug delivery. It can distinguish MUC1-positive cells from MUC1-negative cells by fluorescence imaging. This vector has low toxicity to MUC1-negative cells and can effectively kill adriamycin-resistant BC cells [190]. Therefore, after solving the drawbacks, NPs can also be used as a therapeutic diagnostic for CCA as well as other cancers.

### 5.3. Vaccines

MUC1 is also a promising target for vaccine development, which can induce an immune response against MUC1 [191]. Recently, MUC1 was rated by the American Cancer Institute Working Group as one of the most promising cancer vaccine-targeted antigens in clinical practice [166]. Vaccines targeting MUC1 include subunit, DNA, viral, dendritic cell (DC), and glycopeptide vaccines [192]. The B subunit of Vibrio cholerae toxin (CTB) has great potential as a carrier for subunit vaccines. Pinkhasov et al. ligated the VNTR region of MUC1 with CTB to form a subunit vaccine (CTB-MUC1). The vaccine was then delivered orally to tumor-bearing mice, and they found that the vaccine effectively inhibited tumor growth in the mice [193]. The DNA vaccine (pcDNA3.1-VNTR) developed by Rong et al. can enhance MUC1-induced CTL activity, inhibit tumor growth in mice, and prolong their survival time [194]. Quoix et al. developed a viral vaccine (TG4010), a recombinant viral vaccine targeting MUC1, which significantly improves the survival rate of non-small cell lung cancer (NSCLC) patients with TG4010 treatment [195]. In addition, targeted MUC1 vaccines (DC-based vaccine) based on dendritic cells (DC) have broad therapeutic prospects. Studies have shown that this vaccine can induce anti-tumor immune responses, thereby prolonging the survival of NSCLC patients [196]. Finally, the MUC1 glycopeptide vaccine targeting MUC1-Tn and MUC1-STn has been synthesized, which is used to diagnose BC and PAAD. However, its clinical performance is still being evaluated in phase II/III clinical trials [197].

With the deepening of anti-tumor immune mechanisms, MUC1-based vaccines have become a major concern in the clinical diagnosis and treatment of cancer. Cancer vaccines targeting MUC1 have not yet been successfully used in the clinic. However, it must be emphasized that cancer vaccines targeting MUC1 have great anti-tumor potential when further refined and evaluated in clinical trials [192].

### 5.4. CAR-T

A new approach to immunotherapy is the use of chimeric antigen receptor (CAR) T cells that are engineered to effectively target cancer-associated glycans [198]. CAR-T is a type of T cell that can recognize a specific antigen and has the function of killing cancer cells when injected into the patient’s body. This tumor-targeted therapy method is called CAR-T therapy [199]. CAR-T therapy has made promising breakthroughs in the treatment of hematological malignancies, but its therapeutic role in solid tumors is limited [153]. Carl H June’s team in 2016 designed a new CAR-T cell targeting MUC1-Tn. This CAR-T cell inhibits the growth of MUC1-Tn-positive cancer cells in the mouse model of leukemia and PAAD [200]. Then they designed CAR-T cells targeting c-Met and evaluated the safety and feasibility of c-Met-CAR-T cells for the treatment of metastatic breast cancer in a phase 0 clinical trial, and found extensive tumor necrosis at the injection site, tumor cell debris, and good tolerability [201]. Recent studies have indicated that MUC1-Tn is a potential therapeutic target for intrahepatic cholangiocarcinoma (ICC) and that CAR-T cells targeting MUC1-Tn can specifically eliminate MUC1-Tn-positive ICC cells but not MUC1-Tn-negative ICC cells in vitro and in vivo, which may be a novel therapeutic strategy for ICC [200,202]. Meanwhile, CAR-T cells targeting MUC1 (MUC28z CAR-T) exhibited specific cytotoxicity against TNBC, and after recognizing MUC1 on TNBC cells, the MUC28z CAR-T cells markedly inhibited the growth of TNBC cells with minimal damage to normal breast epithelial cells [153].

In fact, the efficacy of CAR-T therapy in solid tumors has always been poor, which may be due to the TME inhibiting the action of CAR-T cells as well as the incomplete activation of T cell function. Therefore, to improve the efficacy of CAR-T cells against solid tumors, Zhang et al. constructed a new CAR-T cell in 2020. They used the JAK-STAT structural domain to provide cytokine signals to CAR-T cells. This enhanced MUC1-CAR-T cell has stronger cytotoxicity and more significant inhibitory effect on esophageal cancer (EC) [203]. In the same year, Shao et al. designed an inverted chimeric cytokine receptor (ICR), which consists of the extracellular structural domain of TGF-β and the intracellular structural domain of the IL-7 receptor, co-expressed on CAR-T cells. It can target prostate-specific membrane antigen (PSMA), which exhibits superior anti-tumor ability and prolongs the survival time of PRAD mice [204]. Based on the above studies, CAR-T cells targeting MUC1 are also expected to make more progress.

Most solid tumors have large numbers of cancer-associated macrophages (TAM) within the TME [205]. Since the TME inhibits the efficacy of CAR-T cells, attention has shifted to macrophages. Recently, CAR-macrophages (CAR-M) have emerged as an alternative therapy [206]. The first-generation CAR-M cells work by targeting specific antigens to recognize cancer cells and increase their phagocytosis. This only used the phagocytosis of macrophages [205]. The second generation of CAR-M cells is under development. In addition to maintaining the properties of the first-generation CAR-M, the goals of the second-generation CAR-M also include enhancing the presentation of tumor antigens and T cell activation [205]. So far, CAR-M is mainly in the preclinical stage and is undergoing a Phase I trial. This is the HER2-based CAR-M (CT-0508) reported by Klichinsky et al. [207]. In vitro experiments have shown that CT-0508 can not only recognize antigens, but also more effectively activate T cells. In vivo experiments have shown that CT-0508 significantly prolongs the survival period of tumor mice and reduces lung metastasis [207]. These data show that CAR-M therapy has great potential in treating cancers, and not only that, it can increase T-cell activity while treating cancers [205]. In the near future, it is promsing for scientists to envisage the application of CAR-T therapy along with the use of CAR-M therapy for adjuvant treatment, which has the high potential to improve the efficacy.

In summary, cancer-targeted therapy based on mucin is a field with rich research, but it is still worth further research. Because of the complex relationship between different mucins, we need to delve deeper into their functions in cancer. Secondly, most of the mucin-based cancer diagnostics are lacking sensitive and specific tools. To develop diagnostic tools with higher sensitivity and specificity, we still need to make substantial improvements in this area. Finally, although mucin glycans are one of the most promising candidate drugs for developing cancer vaccines, their immunogenicity is poor and they cannot trigger effective and long-lasting immune responses. Therefore, the mucin-type antigens need to be coupled with immune activators to achieve a more durable immune response.

**Table 2:** MUC1-based antibodies, radiopharmaceuticals, vaccines, and CAR-T therapies are covered in the text.

Type of Therapy	Designation	Antigenic Epitope/Target	Type of Cancer	Ref.
Antibodies	SM3	PDTRP	BC	[168,169]
	5E10	MUC1-N	PRAD	[170]
	5E5	MUC1-Tn/STn	BC	[171,173]
	TAB004	MUC1(STAPPAHGV)	PAAD	[175]
	hMUC1	MUC1-C	BC	[176]
	HzMUC1	MUC1-N and MUC1-C	PAAD	[177]
Radiopharmac-euticals	^99m^Tc-HYNIC-PR81	MUC1(AVGLSPDGSRGV)	BC	[180]
	DDS	MUC1	TNBC	[181]
	MUC1-FA- [^18^F] SFB	MUC1	BC	[182]
Nanomedicines	PCX/anti-miR-210 NPs	miR-210	CCA	[189]
	MUC1-Td-AS1411	MUC1	BC	[190]
Vaccines	CTB-MUC1	VNTR	BC	[193]
	pcDNA3.1-VNTR	VNTR	PAAD	[194]
	TG4010	MUC1	NSCLC	[195]
	DC-based vaccine	MUC1	NSCLC	[196]
	MUC1-glycopeptide vaccines	MUC1-Tn/STn	BC, PAAD	[197]
CAR-T	Anti-MUC1-Tn-CAR-T	MUC1-Tn	Leukemia, PAAD	[200]
	c-Met-CAR-T	c-Met	BC	[201]
	Anti-MUC1-Tn-CAR-T	MUC1-Tn	ICC	[202]
	MUC28z CAR-T	MUC1	TNBC	[153]
	Enhanced MUC1-CAR-T	MUC1	EC	[203]
	ICR	PSMA	PRAD	[204]
CAR-M	CT-0508	HER2	OV	[207]

## 6. Conclusions and Future Directions

Mucin glycans are involved in the composition of the mucus barrier. The health of the body and homeostasis of the internal environment is dependent on the normal expression of mucin glycans. Abnormal mucin glycan is a significant marker of cancer cells, the phenomenon noted by researchers as early as 1952 [208]. Since then, more and more researchers have devoted themselves to the effect of mucin glycans on the development of tumors. To this day, the mechanisms by which abnormal mucin glycans lead to cancer development have been widely reported. Various pathogenic bacteria and viruses can cause a variety of cancers, but their relationship with mucoglycans has rarely been reported. In the future, we may be able to explore the inter-regulatory roles of mucoglycans and their interactions, which could lead to a more in-depth understanding of the mechanisms of cancer development.

On this basis, targeting mucin glycans turns into a new strategy for cancer diagnosis and treatment. Taking the best-characterized MUC1 as an example, researchers have developed antibodies, radiopharmaceuticals, vaccines, and CAR-T cell therapies against MUC1. However, these drugs and vaccines have some unavoidable shortcomings, and only a small portion have been applied to clinical diagnosis and treatment, which means continuous improvements are needed to increase the sensitivity and specificity of targeted therapies and to minimize their side effects and toxicity to normal cells. There is encouraging news that MUC1 targeting CAR-T cells has been successfully used in clinical trials for the treatment of advanced NSCLC and BC [209]. As we continue to explore the pathogenic mechanisms by which mucin glycans regulate cancer progression, the combination of targeted mucin glycans with existing cancer treatment options may have a significant impact on cancer treatment.

## Figures and Tables

**Table 1:** Clinical treatment strategies based on mucin-targeted cancers.

Type of Mucin		Type of Cancer	Ref.
Secreted mucins	MUC2	Pseudomyxoma peritonei, BC	[145,146]
	MUC5AC	PAAD	[147]
	MUC5B	gallbladder carcinoma	[148]
	MUC6	GC	[149]
	MUC19	Lung cancer	[150]
	MUC7	PRAD	[151]
	MUC9	OV	[152]
Transmembrane mucins	MUC1	TNBC	[153]
	MUC3A	GC	[154]
	MUC4	PAAD	[155]
	MUC12	CRC	[156]
	MUC13	CRC	[157]
	MUC16	OV	[158]
	MUC17	myxofibrosarcoma	[159]
